# Supplementary material for: Colorectal Cancer Stem Cells Fuse with Monocytes to Form Tumour Hybrid Cells with the Ability to Migrate and Evade the Immune System
Source: Cancers (Basel). 2022 Jul 15;14(14):3445. doi: 10.3390/cancers14143445 (PMC9324286; doi:10.3390/cancers14143445)
Supplement: Supplementary file 1 [file cancers-14-03445-s001.zip › cancers-1809475-supplementary.pdf]

Supplementary Materials: Colorectal Cancer Stem Cells Fuse with Monocytes to Form Tumour Hybrid Cells with the Ability to Migrate and Evade the Immune System

Karla Montalbán-Hernández, Ramón Cantero-Cid, José Carlos Casalvilla-Dueñas, José Avendaño-Ortiz, Elvira Marín, Roberto Lozano-Rodríguez, Verónica Terrón-Arcos, Marina Vicario-Bravo, Cristóbal Marcano, Jorge Saavedra-Ambros, Julia Prado-Montero, Jaime Valentín, Rebeca Pérez de Diego, Laura Córdoba, Elisa Pulido, Carlos del Fresno, Marta Dueñas and Eduardo López-Collazo

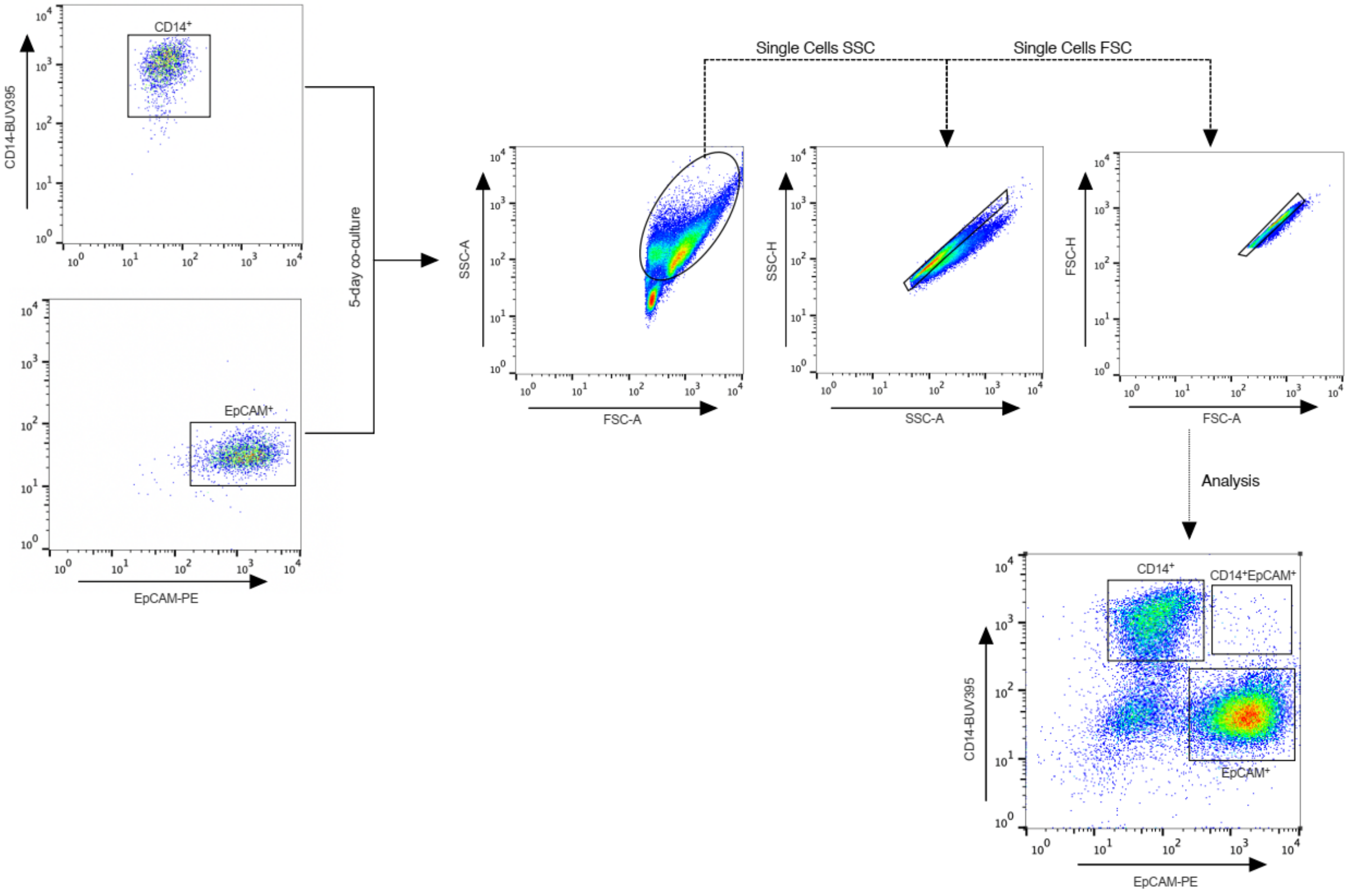

Figure S1. Gating strategy of in vitro co-culture THC identification.

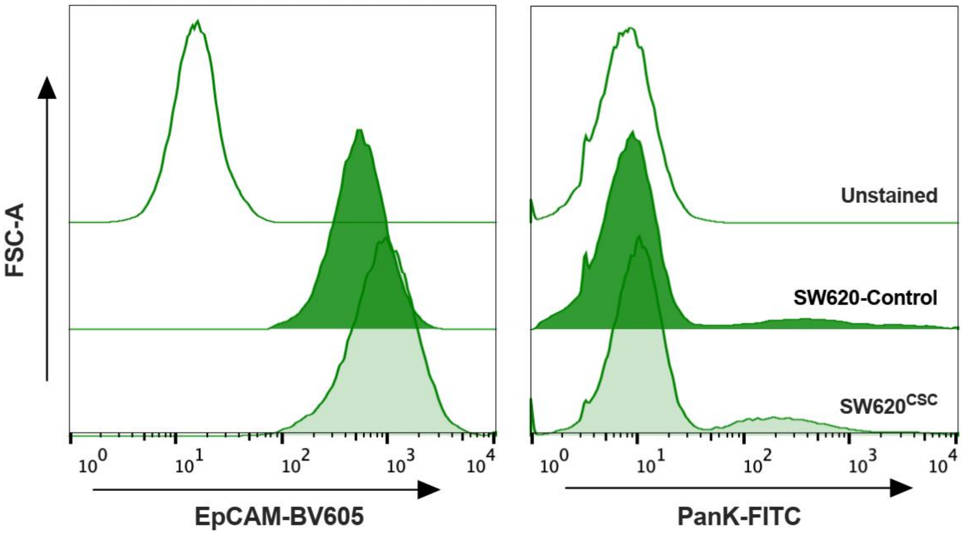

Figure S2. Fluorescence intensity of EpCAM (left) and PanK (right) markers on SW620-control and SW620CSCs.

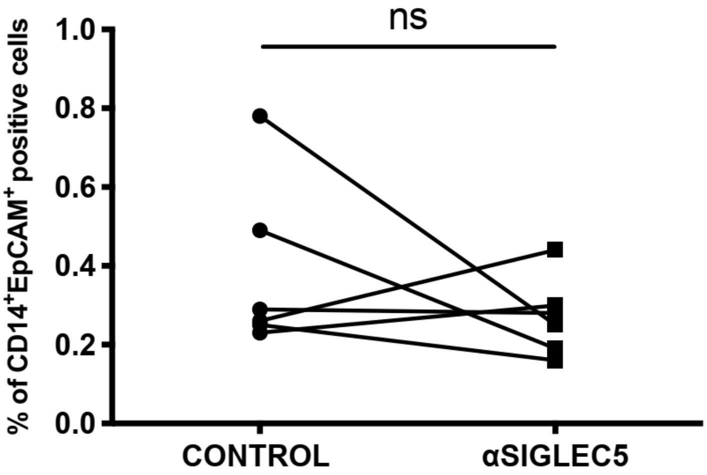

Figure S3. Blockade of anti-SIGLEC5 has no effect on the number of THCs generated.

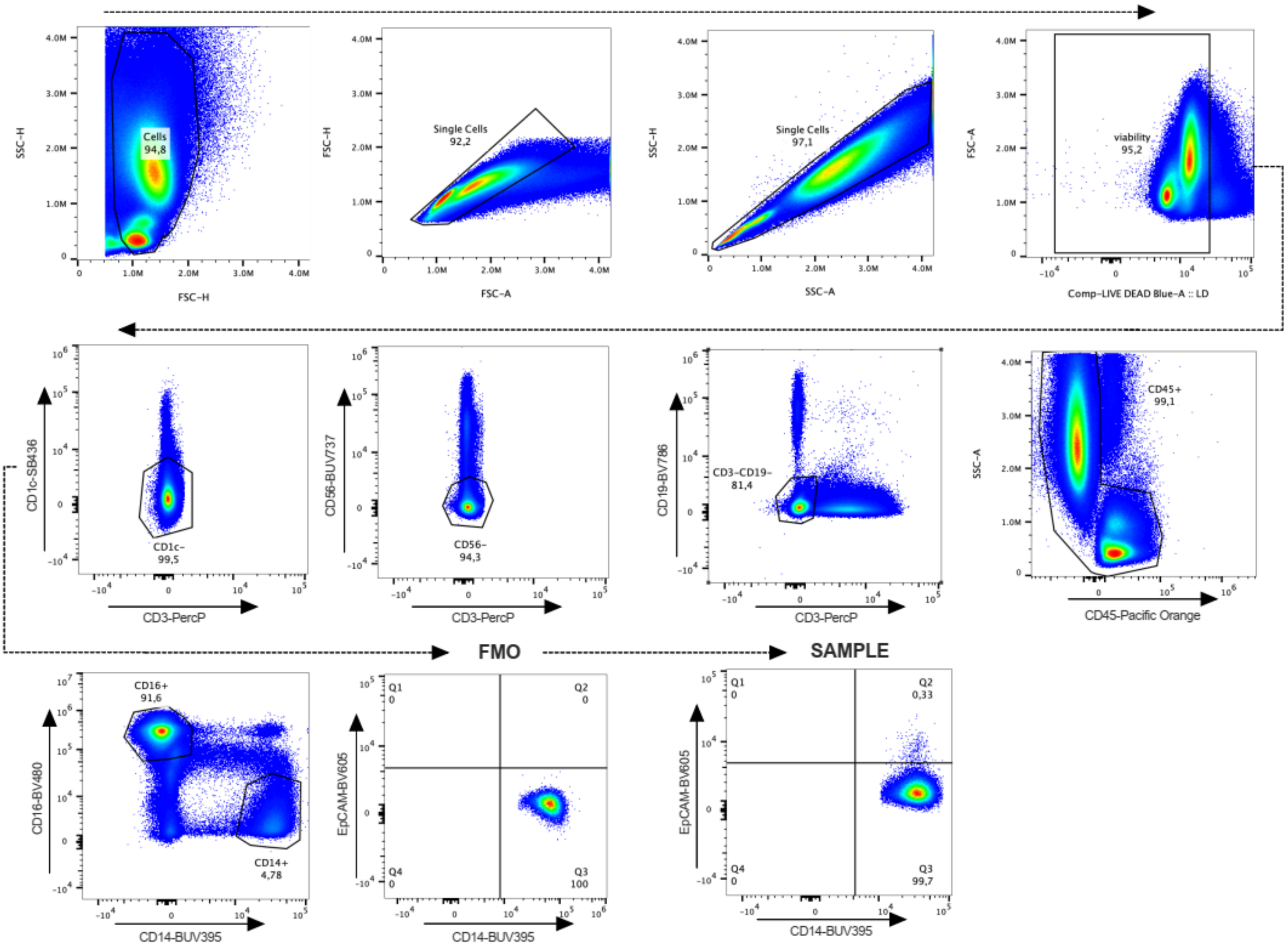

Figure S4. Gating strategy for THC identification on whole blood samples of CRC patients.

Table S1. Sequence of primers used.

Gene	Primer	Sequence (5′-3′)
Beta-Actin	Forward	GTG GGG CGC CCC AGG CAC CA
	Reverse	CTC CTT AAT GTC ACG CAC GAT TTC
KLF4	Forward	ACC CAC ACA GGT GAG AAA CC
	Reverse	ATG TGT AAG GCG AGG TGG TC
NANOG	Forward	TGA ACC TCA GCT ACA AAC AGG TG
	Reverse	AAC TGC ATG CAG GAC TGC AGA G
SOX2	Forward	AGAACCCCAAGATGCACAAC
	Reverse	CGGGGCCGGTATTTATAATC
c-MYC	Forward	GCCAAGCTCGTCTCAGAGAAG
	Reverse	CAGAAGGTGATCCAGACTCTG
OCT3/4	Forward	CTTGCTGCAGAAGTGGGTGGAGGA
	Reverse	CTGCAGTGTGGGTTTCGGGCA
